# Leg dominance and performance in change of directions tests in young soccer players

**DOI:** 10.1038/s41598-022-17245-5

**Published:** 2022-07-28

**Authors:** Filipe Manuel Clemente, Francisco Tomás González-Fernández, Gabriel García-Delgado, Rui Silva, Ana Filipa Silva, Hadi Nobari, Moisés Falces-Prieto

**Affiliations:** 1 Escola Superior Desporto e Lazer, Instituto Politécnico de Viana do Castelo, Rua Escola Industrial e Comercial de Nun’Álvares, 4900-347 Viana do Castelo, Portugal; 2 Instituto de Telecomunicações, Delegação da Covilhã, 1049-001 Lisboa, Portugal; 3 Department of Physical Education and Sports, Faculty of Education and Sport Sciences, Campus of Melilla, University of Granada, 52006 Melilla, Spain; 4 Department of Physical Education and Sports, Pontifical University of Comillas. CESAG, 07013 Palma, Spain; 5 The Research Centre in Sports Sciences, Health Sciences and Human Development (CIDESD), 5001-801 Vila Real, Portugal; 6 N2i, Polytechnic Institute of Maia, Maia, Portugal; 7 Department of Exercise Physiology, Faculty of Educational Sciences and Psychology, University of Mohaghegh Ardabili, Ardabil, 56199-11367 Iran; 8 Department of Motor Performance, Faculty of Physical Education and Mountain Sports, Transilvania University of Braşov, 500068 Braşov, Romania; 9 Faculty of Sport Sciences, University of Extremadura, 10003 Cáceres, Spain; 10 Sepahan Football Club, Isfahan, Iran; 11 Research Center High Performance Soccer, Marcet Academy, 08035 Barcelona, Spain

**Keywords:** Physiology, Medical research, Engineering

## Abstract

The present study aimed to examine the influence of leg dominance on the change of direction (COD) performance. In this study participated 94 healthy young highly trained male soccer players belonging from two categories (n = 27 vs. n = 67; 14.81 ± 0.40 vs. 16.64 ± 1.25 years of age; 170.61 ± 5.61 vs. 173.73 ± 7.19 cm of height; 64.74 ± 8.44 vs. 66.70 ± 7.95 kg of weight, for U16 and U18, respectively). Fitness assessments were performed two times in a period of three months, and included: (1) anthropometry measures, (2) 30–15 IFT, (3) 10-m sprint test, (4) 505-COD test, 90° COD test and cross-over hop test. A paired sample t-test was performed to evaluate the asymmetries at the intragroup level in each of the COD’s tests. A symmetry index was used to analyse the asymmetries between categories, and an independent sample t-test was used to compare the variability between the two categories in each of the three tests performed. The effect size was also evaluated. Analysis demonstrated that evidence a trend for a better performance with the preferred leg in the cross-over hop and 505-COD tests, and with the non-dominant leg in the 90° COD. However, in the intragroup analysis, only the 505-COD test registered differences, and no differences were notice din the intergroup comparison. Only in the 505-COD test the percentage of variability (CV) was statistically significant (7.03 ± 4.18% vs. 4.03 ± 2.02% from U16 and U18, respectively). In sum, bilateral differences were noticed in the intragroup comparison, although only in 505-COD test the leg dominance showed to influence performance. In the intergroup analysis any difference was noticed between age categories.

## Introduction

Change-of-direction (COD) ability assumes an importance role in invasion team sports, during both training and competition, as it is one of the most determinants of success in the final outcomes of this type of sport competition^1^. In fact, among the linear sprinting actions, which is considered the most physical capacity performed in goal situations, soccer players can reach up to approximately 700 COD actions at different angles and intensities throughout a single soccer match that can also influence the final outcome of a match, especially in set pieces^2^.

Invasion team sport athletes produce high-intensity actions mainly through unilateral lower-limbs movement patterns^3^. Also, the majority of players have a preferred limb (i.e., dominant leg)^4^. Indeed, team sport athletes seem to use their dominant leg to perform the majority of technical actions, such as ball control, passing, shooting, crossing, and even to perform COD tasks^5^. Given that, it is expected a higher volume of actions executed with the dominant leg than with the non-dominant leg, which induces some levels of lower limb asymmetries in terms of strength^6^. Although these lower limb strength asymmetries seem to decrease player’s technical and physical performance, there is still incongruences in the available literature regarding their cause-effect^7,8^.

There are several COD tests available to be conducted on athletes during periodic assessment during the season^9,10^. In practice, coaches and practitioners during a testing battery usually conducts one COD test that they feel that is better suited to their soccer teams^10^. In this sense, it is imperative to investigate the influence of leg dominance during different COD tests. Each of the available COD tests has their own characteristics. For example, the cross-over hop test (CHT) involves a jumping action from side-to-side from a static position, while the 505-COD test and de 90° COD tests involves running, in which both acceleration and linear sprint are present. Also, the 505-COD test involves only one COD, while the 90° COD test involves three CODs.

Furthermore, in 505 and 90° COD tests, the time to complete the test is used as the final outcome. However, the presence of acceleration and linear sprint in such tests can produce bias in the final outcomes. That is, players with greater acceleration and linear sprint capacity can outperform their slower counterparts and still, be worse at changing direction^9^. For those reasons, it seems prudent to consider other tests that does not have the influence of neither acceleration nor linear sprint, to assess lower limb asymmetries during COD tasks, and to analyze their differences. Moreover, in a study conducted on 73 youth soccer players, it was found that in performing a sidestepping COD test with the dominant leg was significantly better than with the non-dominant one. While, during other COD test with different angles, the dominant leg was better only during a 135° COD^13^. In fact, leg differences can produce different outcomes obtained from COD tests, which is also associated with functionality and non-functionally symmetries occurring in athletes^14–16^.

Beyond all the above-mentioned, players form different age categories can also perform differently during COD tasks and during different COD tests, as older players usually generate greater force levels in acceleration and sprint phases^17^. On the other side, the motor awkwardness of maturing players can be detrimental during COD tasks^18^. However, there are few studies analysing lower limb asymmetry during different COD tests in youth soccer players. A recent study conducted on 68 youth soccer players, found greater lower limb asymmetries during the 90° COD test for U-16 than the U-15, U-17 and U-18 age categories. Therefore, the aim of the present study was to examine the influence of leg dominance on the change of direction performance.

## Methods

### Experimental approach to the study

Participants completed 3 testing sessions, 7 days apart. During each session, we collected data from a cross-over hop test, 505 and the 90° COD test. Participants were familiar with all the tests as they were part of their regular fitness testing battery. Both testing sessions were completed at the same time of the day and during participants’ regular training times and always before training. This study was conducted in May of 2020/2021 season and consisted in a weekly resistance training session on day-4 (Wednesday), allowing a rest of 72 h prior to match and within the usual training hours (15:30–18:00 h). The assessments were carried under weather conditions (29 °C and 50% humidity). The type of grass was synthetic and of the 3rd generation basically characterized by being composed of the following elements: a support fabric and fibers woven to the support with a height between 35 and 70 mm and with a distance between them that allows the incorporation of a filling.

#### Participants

A total of 94 young highly trained male soccer players from high-performance academy were part of the present study. The soccer players were assigned into two categories: under 16 (U16) [(n = 27); age: 14.81 ± 0.40 years; height: 170.61 ± 5.61 cm; weight 64.74 ± 8.44 kg)] or under 19 (U18) [(n = 67); age: 16.64 ± 1.25 years; height: 173.73 ± 7.19 cm; weight 66.70 ± 7.95 kg)]. All participants were familiar with the evaluations carried out and they had experience in the different test carried out. These players trained 9 h of soccer training and played one competitive match per week.

Each soccer players and respective parents were informed about the objective of present study and giving the signed consent before the beginning of the study. The inclusion criteria were as follows: (1) being an active player with federation license with previous experience of ≥ 5 years (2) not presenting any injuries during the previous two month; (3) belong in the academy a full season; (4) participating in 3 tests proposed (100%) and (5) giving consent.

The participants were treated according to American Psychological Association (APA) guidelines, which ensured the anonymity of participants’ responses. In addition, the study was conducted in accordance with the ethical principles of Helsinki declaration^16^ for human research and was approved by the Research Ethics Committee of the Pontifical University of Comillas (internal project n°: 2021/65).

#### Procedure

**Figure 1:**
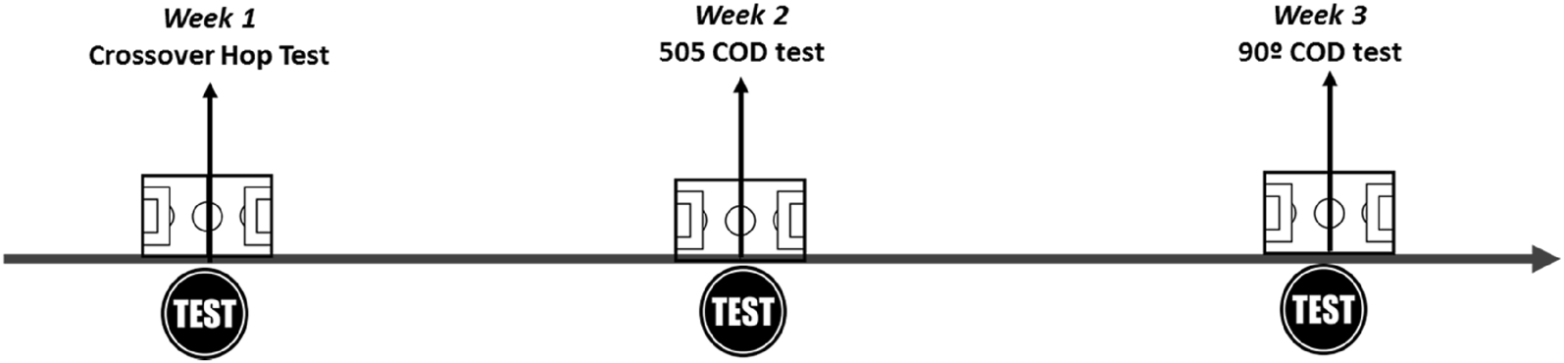
Schematic representation of a test day (see text for full description).

Prior to conducting any tests, participants conducted a standardized warm up consisting of low aerobic activity, dynamic stretching, progressive sprinting, and submaximal pre-planned changes of direction, lasting 10 min. Following the standardized warm up, participants received verbal instruction and demonstrations from the research team immediately prior to conducting 2 familiarization attempts for each test. Recovery intervals between attempts were standardized at three minutes for each test. For the selection of the dominant leg, the players were asked which leg they preferably use to control, pass and throw the ball regardless of playing position^20^. All the evaluations (see Fig. 1) were performed at the same time and space, with the usual clothing for the soccer player, the specific footwear and supervised by the same technical specialists.

#### Measures

##### Cross-over hop test

**Figure 2:**
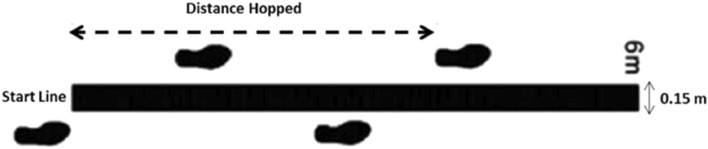
Set up for the cross-over hop test.

For the cross-over hop test (CHT), the participant performed three consecutive jumps over a 15 cm line that had been marked on the floor. The test consists of doing three jumps starting from a monopodal support and landing with the same leg as the impulse^21^. Subjects were instructed to place their hands on their hips and to maintain the landing position for 3 s (sec), without loss of balance or performing additional movements involving the free limb. The distance reached was measured in centimeters from the take-off line to the heel in the final position^21^. They made two attempts and the best was selected. See Fig. 2.

##### 505 COD test

**Figure 3:**
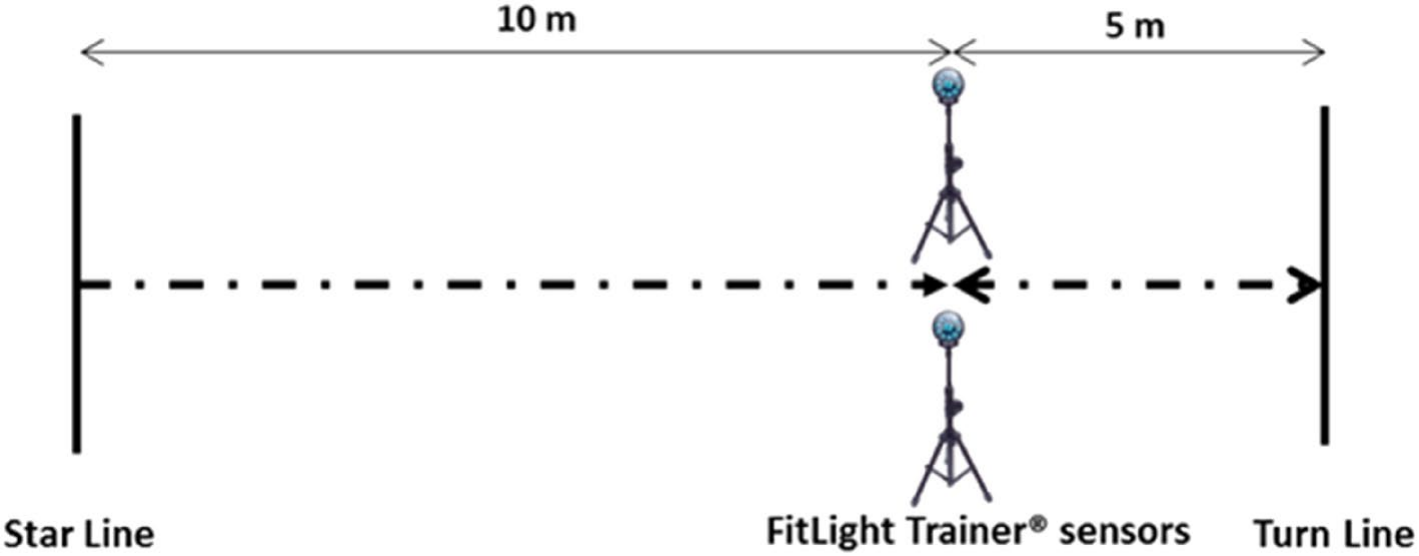
Set up for the 505 COD test.

The methodology for the 505-COD test was as per originally established methods^22^, see Fig. 3. Therefore, this involved a 10 m linear sprint from a static start, a 180° turn using the right or left leg to stop the run (ensuring contact with a designated line), and a 5 m return sprint through an identified finish line. The time taken to complete the final 5 m of the 10-m linear sprint, turn, and 5 m return sprint was recorded^20^. For speed evaluation, 2 attempts were performed with a recovery time of 2 min between repetitions and an average of the two repetitions for subsequent analysis^24,25^. Times were measured in sec. The evaluation system was carried out through FitLight Trainer^®^ sensors (Ontario, Canada). This system is used to calculate agility, reaction time, speed and coordination in football^26^. The optoelectronic devices were adjusted to an appropriate hip height as per the mean stature of the sample group. The recorded time for each of the players was stored in a portable tablet with an Android system and its subsequent analysis in the Microsoft Windows^®^ Excel program (Redmond, Washington, USA).

##### 90° COD test

**Figure 4:**
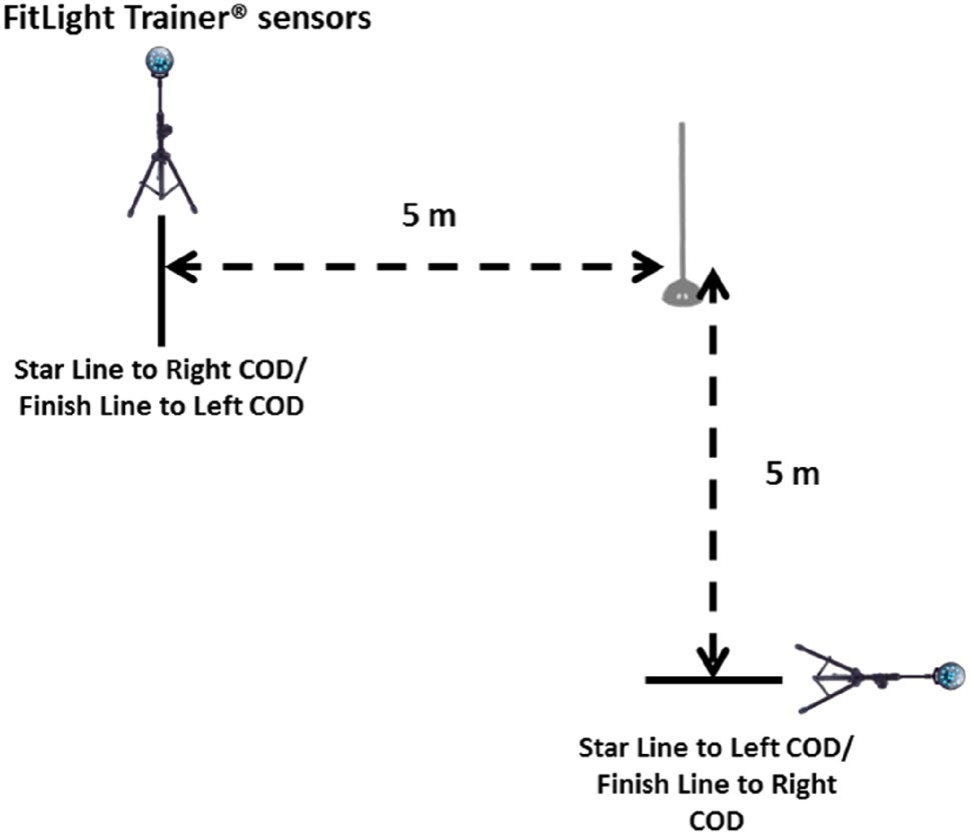
Set up for the 90° COD test.

Finally, they performed three 90° COD test (right/left) (10 m) with 90° (COD). See Fig. 4. The times of each repetition were taken in seconds. For data analysis, the average of the two attempts made in each series with 2 min of recovery among them was chosen^24,25^. For the evaluation, 2 Led FitLight Trainer^®^ sensors were placed, one at the beginning and the other at the end of the route. Timing gates were adjusted to an appropriate hip height as per the mean stature of the sample group.

### Statistical analysis

Data were analyzed using software IBM SPSS Statistics (version 26). For data processing, mean and standard deviation were used. The variability of the results in each test [(percentage of variability (CV)] was also computed having into account the totality of the test performed per subject for each test (two to the dominant side and two to the non-dominant side). To evaluate the asymmetries at the intragroup level in each of the COD’s tests, a t-test of paired samples was performed, comparing the time of the test depending on different criteria (to facilitate the understanding of the results).

- In the test CHT the criterion was the leg used to perform the jumps (dominant or non-dominant).
- In the test 505 COD test the criterion was the legs that performs the last support before changing direction (dominant or non-dominant).
- In the 90° COD test the criteria was the turning direction (dominant or non-dominant).

To study the asymmetries between U16 and U18 in the CHT, the symmetry index (SI) in percentage was calculated^27^ using different formulas, based on the mentioned criteria’s: the formula (1) was used for the cross-over hop test (SI-CHT), the formula (2) was used for the 505 COD test (SI-505) and formula (3) was used for the 90° COD test (SI-90). Then a t-test between U16 and U18 players was performed for independent samples using the symmetry index as the dependent variable

1 $$SI-CHT \left(\%\right)=\frac{({H}_{d}-{H}_{nd})}{0.5\times ({H}_{d}+{H}_{nd})}\times 100;$$

where H_d_ is the time of the COD test performing the hop with the dominant leg and H_nd_ the time performing the hop with the non-dominant leg

2 $$SI-505 \left(\%\right)=\frac{({S}_{d}-{S}_{nd})}{0.5\times ({S}_{d}+{S}_{nd})}\times 100;$$

where S_d_ is the time of the COD test performing the stop with the dominant leg and S_nd_ the time of the COD test performing the stop with the non-dominant leg

3 $$SI-90 \left(\%\right)=\frac{({H}_{d}-{H}_{nd})}{0.5\times ({H}_{d}+{H}_{nd})}\times 100;$$

where T_d_ is the time of the COD test performing the turn toward the dominant side and T_nd_ the time of the COD test performing the turn towards the non-dominant side.

Finally, an independent sample t-test was used to compare the variability (CV [%]) between the two age categories in each of the three tests performed. Significant p value was established at 0.01. The effect size was evaluated using the Cohen scale^28^: (1) 0–0.20, negligible effect; (2) 0.20–0.50, small effect; (3) 0.50–0.80, medium effect; (4) 0.80–1, large effect.

### Ethics approval and consent to participate

Players and their parents or guardian were then invited to sign an informed consent document before any of the tests were performed. The study has conducted according to the Declaration of Helsinki, and the study was conducted in accordance with the ethical principles of the 1964 Helsinki declaration for human research and was approved by the Research Ethics Committee of the Pontifical University of Comillas (2021/65).

### Consent for publication

Authors have a formal written consent of each participant, in order to publish data after the manuscript may be accepted.

## Results

Descriptive data showed a predominance of players who performed better jumping with the preferred leg in the case of the CHT or stopping with the dominant leg in the case of the 505 COD test (See Table 1, for more information). In the case of the CHT the numbers of players who obtained a better result jumping with the dominant leg was 61 versus 43 who performed better jumping with the non-dominant leg. In the 505 COD test the number of players who performed better stopping the sprint with the dominant leg was of 64 versus 30 who performed better stopping with the non-dominant leg. In the 90° COD test, 39 players turned faster towards the dominant side and 54 towards the non-dominant side.

**Table 1:** Descriptive values of the symmetry index by COD test and by best hopping leg (cross-over hop test), best braking leg during cutting (505 COD test) or best turning direction (90° COD test).

Test	Best leg/direction	Group	N of players	SI (%)	SI SD (%)	SI p25th (%)
Cross-over hop test	Best hopping leg (D)	U16	15	6.6	5.2	10.1
		U18	36	5.6	4.1	8.9
		All	51	5.9	4.4	9.1
	Best hopping leg (ND)	U16	12	5.9	4.3	9.7
		U18	31	5.3	4.5	6.8
		All	43	5.5	4.4	2.4
505 COD test	Best braking leg (D)	U16	17	7.8	7.7	11.2
		U18	47	4.2	2.7	5.5
		All	64	5.2	4.8	6.4
	Best braking leg (ND)	U16	10	4.6	3.7	6.7
		U18	20	4.1	2.7	6.3
		All	30	4.3	3.0	1.8
90° COD test	Best turning direction (D)	U16	8	5.7	4.2	8.9
		U18	31	4.6	3.7	5.8
		All	39	4.8	3.8	6.3
	Best turning direction (ND)	U16	19	6.7	4.6	12
		U18	35	4.3	4.2	6.8
		All	54	5.2	4.5	1.6

*D* dominant, *ND* non-dominant.

**Figure 5:**
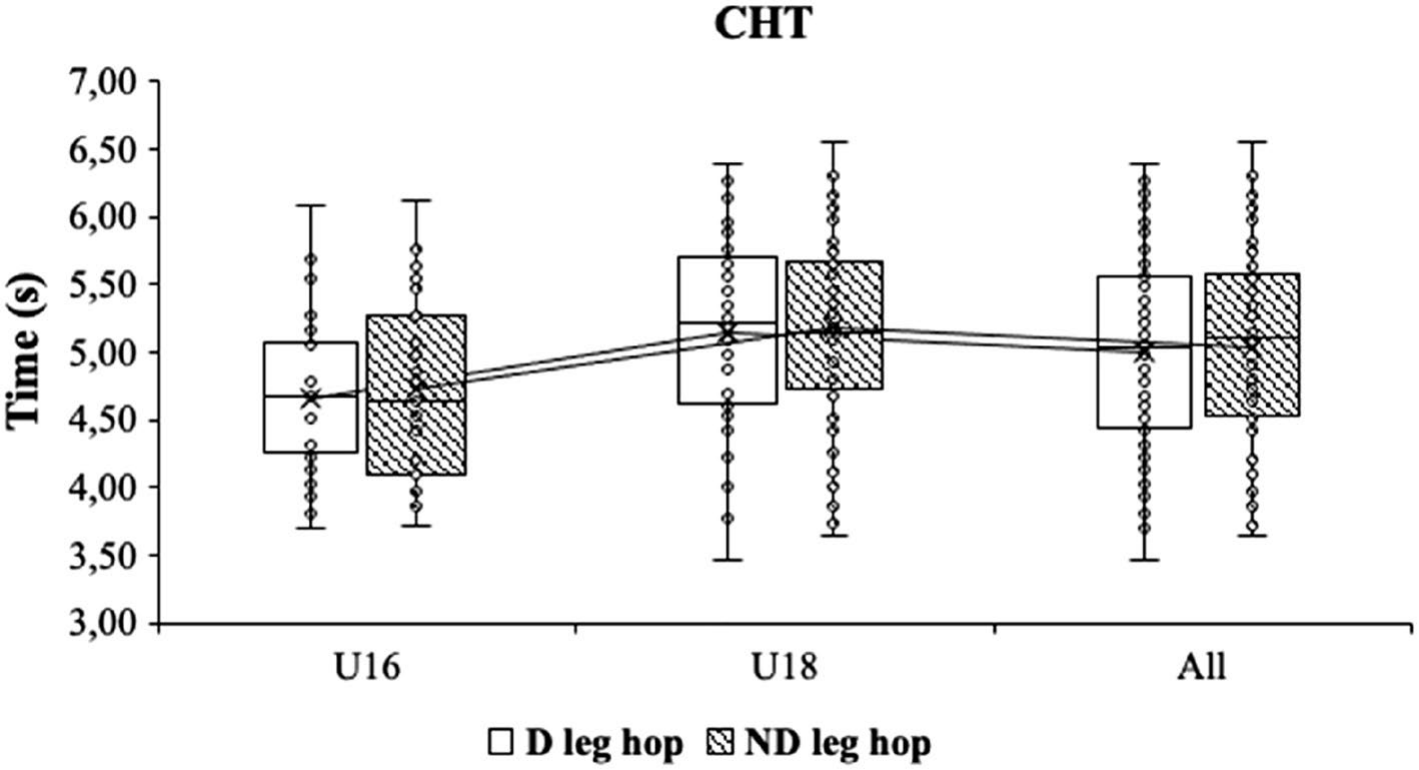
Intragroup comparisons of the change of direction text. *CHT* cross-over hop test, *D* dominant, *ND* non-dominant.

**Figure 6:**
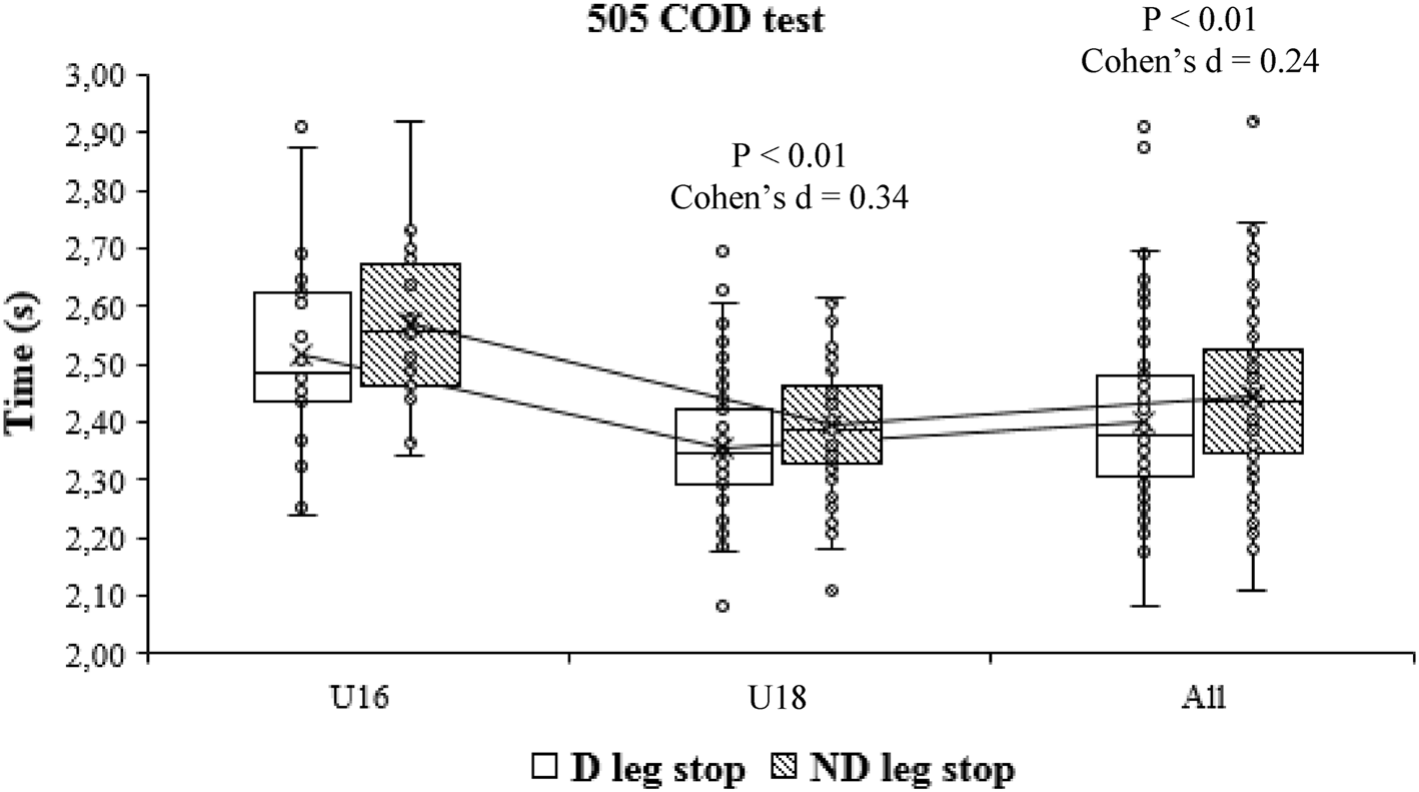
Intragroup comparisons of the 505 COD test. *D* dominant, *ND* non-dominant.

**Figure 7:**
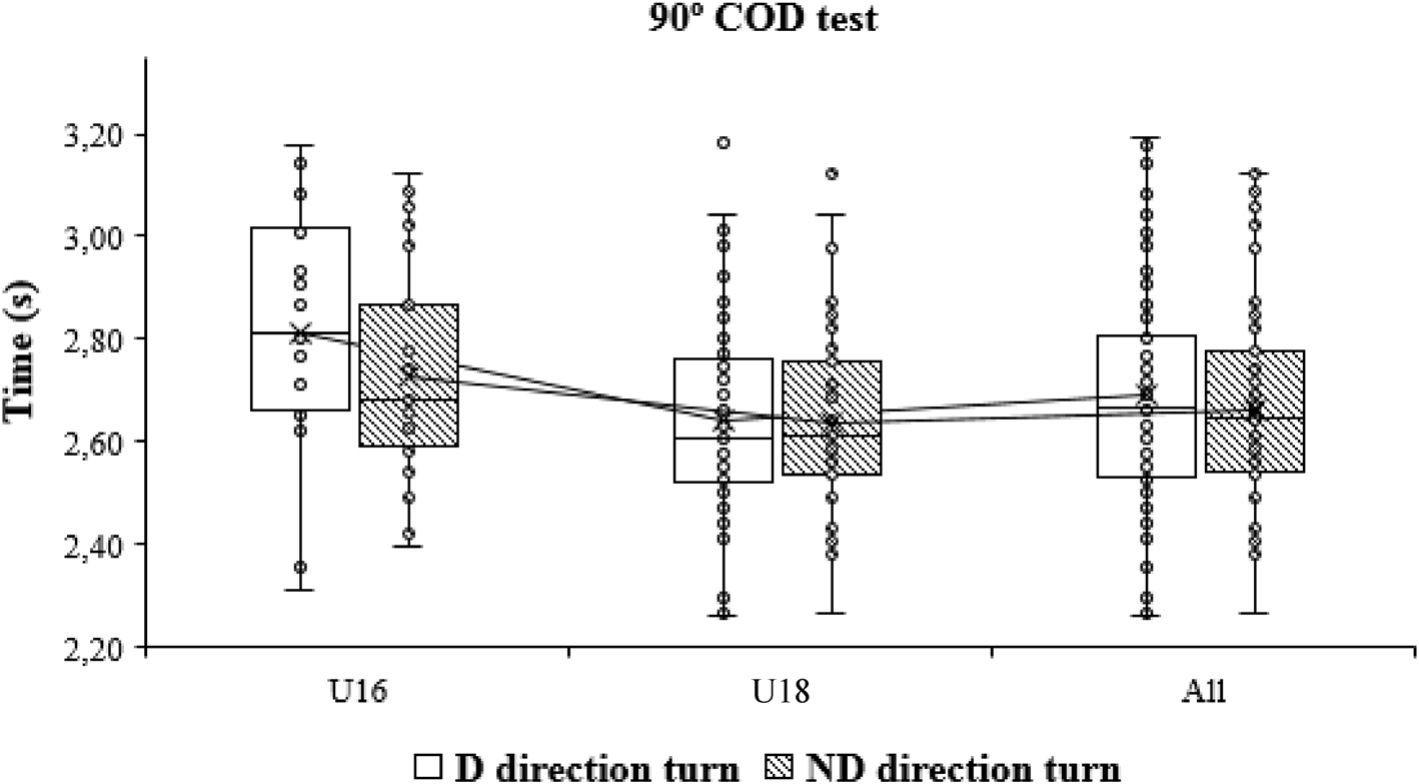
Intragroup comparisons of the 90° COD test. CHT. *D* dominant, *ND* non-dominant.

When the time of the performance of the COD test was compared at the intragroup level depending on the dominance, differences were only obtained in the case of the 505 COD test, with the players being faster when they stop with the dominant leg (2.39 ± 0.17 s vs. 2.44 ± 0.14 s; p < 0.01) (Fig. 5) when they stop with the dominant leg, when the full sample was analysed and in the case of the U18 players. The effect size in this case was small (Cohen’s d = 0.35). Posteriorly, intragroup comparisons of the 505 COD test. D: Dominant; ND: non-dominant (Fig. 6). In addition, intragroup comparisons of the 90° COD test. CHT. D: Dominant; ND: non-dominant (Fig. 7).

**Figure 8:**
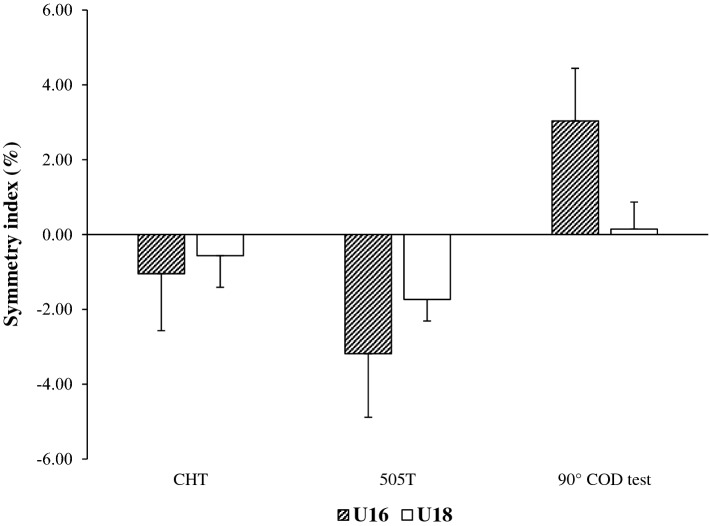
Intergroups comparisons of the symmetry index by age category in the change of direction tests. *CHT* cross-over hop test, 505 COD test and 90° COD test.

In the case of intergroup comparisons, although the U16 had higher values of asymmetries than the juveniles in the three COD tests, there were no significant differences in any of the cases analysed (Fig. 8).

Relatively to the variability values of the test performed, the CV was of 7.07 ± 3% (U16) and 5.99 ± 3.49% (U18) in the CHT, of 7.03 ± 4.18% (U16) and 4.03 ± 2.02% (U18) in the test 505 COD test and of 5.7 ± 3.33% (U16) and 4.82 ± 2.25% (U18) in the 90° COD test. There were statistically significant differences only in the case of the 505 COD test between the two age categories, the effect size being large (p < 0.001; Cohen’s d = 1.07).

## Discussion

The results of the intragroup comparisons showed bilateral differences in each test. A greater number of subjects performed the test better by jumping with the dominant leg in the case of the cross-over hop test or braking with the dominant leg in the case of the 505 COD test. In the case of the 90° COD test, a greater number of subjects performed better when they turned in the direction of the non-dominant leg. Despite this, only in the case of the 505 COD test these differences were significant. Intergroup comparisons did not show significant differences between groups U16 and U18, in any of the three tests evaluated.

Being asymmetric in human activities is part of biology since sports performance and sports training conducts to an overuse of a specific leg for performing specific actions^29^. Thus, asymmetry is somehow part of human biology with some benefits if functional^30^. This functionality is highly individual since for some players a huge asymmetry can be a support for better performance, while for another one may be detrimental^31,32^. In our study was found that 59% of the best results come from dominant leg, while 68% of the best results in 505 COD test was obtained in the dominant leg. This confirms an evidence that COD is typically performed in a faster way using dominant leg^13^. In the opposite way, only 42% of the best results in 90° COD test was observed in the dominant leg.

The results of the current research revealed that in an intra-group analysis, in particular U18 group, performing COD in 505 COD test with dominant leg (in case the preferred leg) helped players to be significantly faster while using the leg for braking. These findings are in line with previous research conduct in soccer players^33,34^. This can be partially explained by different strength levels occurring in preferred and non-preferred legs regarding knee flexors/extensors and hip abductors/adductors^13^. As example, in braking hip joint will play a determinant role while knee extensors and flexors may help to eccentrically decelerate the downward motion of the bod’s centre of mass to stabilize the knee joint and also provide a support for the propulsion phase^13^. Thus, considering that strength levels can be different between legs (in some cases greater than 14%)^35^, it can be expectable observe a consequence in COD performance between legs as well.

Regarding between-age group analysis, only significant differences were found in 505 COD test (best results in older). Since COD time in 505 COD test is predominantly explained by acceleration^36^ and considering that greater force and sprint are observed in latest stage of youth period^37^, it is expectable to observe the results observed in this test. This also highlight that besides typical field-based training, physical qualities related with COD tests must be oriented to improve the skill across the time. Interestingly, the asymmetries were not significant different across age groups, which is not in line with a previous study testing different age groups asymmetries COD tests performed in soccer^34^. However, in such a study^34^, COD deficit was considered and not properly the COD time which means a significant difference in the type of outcomes. The COD time express a great dependency of acceleration, thus smaller variations in COD ability, while COD deficit is a better representation of COD ability with less dependency from sprint.

### Practical application

It is well known that asymmetry is a detrimental factor to soccer performance. However, there are some doubts about the practical evidence on soccer and the type of asymmetry because is often not defined. Crucially, the majority of soccer asymmetries are a results of limb dominance and increase with the training and competition. For this reason, testing the existence of asymmetries on soccer, that in the present work are linked with COD test, could be effective in detecting sporting asymmetries. Therefore, professional and multidisciplinary (physical coach, physical therapist and medical staff) training interventions can reduce the different sporting asymmetries and improve the soccer performance. In fact, observe the different sporting asymmetries in different categories could be a decisive strategy in light of ‘development windows’ that can be targeted for attaining more general neuromuscular improvements^26^.

## Conclusions

As a conclusion, we could assume that bilateral differences exist in the whole group of youth soccer athletes, with a greater number of subjects performed the test better by jumping with the dominant leg in the case of the cross-over hop test and in braking with the dominant leg in the case of the 505 COD test. Nevertheless, only in 505 COD test the leg dominance showed to influence performance. When comparing age categories (U16 vs. U18), no difference was noticed in any of the three tests.

## Data Availability

The datasets generated during and analyzed during the current study are available from F.T.G.F. on reasonable request.

## Abbreviations

COD: Changes of direction
CH: Cross-over hop test
APA: American Psychological Association
SI: Symmetry index (SI)
D: Dominant
ND: Non-dominant
